# Large scale across-breed genome-wide association study reveals a variant in *HMGA2* associated with inguinal cryptorchidism risk in dogs

**DOI:** 10.1371/journal.pone.0267604

**Published:** 2022-05-26

**Authors:** Matthew Blades, Jamie Freyer, Jonas Donner, Rebecca Chodroff Foran, Oliver P. Forman

**Affiliations:** 1 Wisdom Panel Research Team, Wisdom Panel, Kinship, Waltham on the Wolds, Leicestershire, United Kingdom; 2 Wisdom Panel Research Team, Wisdom Panel, Kinship, Vancouver, Washington, United States of America; 3 Wisdom Panel Research Team, Wisdom Panel, Kinship, Helsinki, Finland; USP FZEA: Universidade de Sao Paulo Faculdade de Zootecnia e Engenharia de Alimentos, BRAZIL

## Abstract

Cryptorchidism is the most common congenital sex development disorder in dogs. Despite this, little progress has been made in understanding its genetic background. Extensive genetic testing of dogs through consumer and veterinary channels using a high-density SNP genotyping microarray coupled with links to clinical records presents the opportunity for a large-scale genome-wide association study to elucidate the molecular risk factors associated with cryptorchidism in dogs. Using an inter-breed genome-wide association study approach, a significant statistical association on canine chromosome 10 was identified, with the top SNP pinpointing a variant of *HMGA2* previously associated with adult weight variance. In further analysis we show that incidence of cryptorchidism is skewed towards smaller dogs in concordance with the identified variant’s previous association with adult weight. This study represents the first putative variant to be associated with cryptorchidism in dogs.

## Introduction

Cryptorchidism, or the failure of one or both testes to descend into the scrotal sac during maturation, is the most common disorder of sex development in dogs, with a reported prevalence of 0.8–10% [1]. Studies indicate cryptorchidism is a moderately heritable trait with an estimated monogenic model heritability of 0.23 in the Boxer dog [2] and is around 2.7 times more common in small breed dogs [1] with some breeds being overrepresented, providing further evidence of heritability. Studies have indicated that average litter sizes in the pig and dog are significantly increased for matings between individuals that have previously produced cryptorchid offspring, suggesting a mechanism by which cryptorchidism is maintained in animal populations [3].

Right-sided inguinal cryptorchidism has been shown to be the most common presentation [4], followed by right-sided abdominal cryptorchidism. Bilateral cryptorchidism is uncommon. Due largely to the increase in temperature associated with an inguinal or abdominal location, cryptorchid testicles are unable to produce sperm. Additionally, the rate of testicular tumors has been shown to be approximately 13 times higher in the cryptorchid testicle than in descended testicles [1]. Testicular neoplasia is one of the most common types of cancer in intact male dogs, with the highest frequency tumor types being seminomas, interstitial cell tumors, and Sertoli cell tumors [5]. Sertoli cell tumors are the least common tumor type of the three in unaffected dogs, but the most common type in cryptorchid testicles, with a relative risk of 9.2 [6]. While many testicular neoplasms are benign, including the majority of Sertoli cell tumors, the increase in endogenous estrogen produced by neoplastic Sertoli cells can cause a syndrome of feminization and bone marrow suppression, which can be life threatening [7, 8]. Testicular torsion, a painful condition requiring emergency surgery, is also seen at a higher frequency in cryptorchid dogs. While the general treatment for cryptorchidism in veterinary patients is removal of both testicles, this may be problematic in breeds predisposed to the disorder, as elimination of these individuals from the breeding population may result in an unintended loss of genetic diversity.

Cryptorchidism is also one of the most common congenital defects in boys, and similar effects on fertility and cancer rates can be seen in cryptorchid men [9]. Cryptorchidism has also been noted as a component of numerous congenital syndromes including Down Syndrome, Noonan Syndrome, and Testicular Dysgenesis Syndrome, in which cryptorchidism is associated with additional defects such as hypospadias and shortened anogenital distance [10]. Though the numerous anatomic and embryologic differences between rodents and humans limit the value of mouse models for study of human cryptorchidism, the dog is one of the few species that is regarded as a suitably similar animal model [11]. However, the genetic basis of cryptorchidism in the dog is not fully understood.

Cryptorchidism has been studied in many species, and multiple genetic associations have been investigated. In humans, the effects of various genes implicated in testicular descent have been studied, including androgen receptor and anti-Mullerian hormone genes [12]. In canines, associations have been made with *ADAMTS20*, *MID1IP1*, *MMP9* (all via GWAS), *COL2A1* (via PCR association study), *HSP70* (via biochemical activity assay), and *INHA* (via expression study) [11]. A GWAS for cryptorchidism in the Siberian Husky identified six putative genomic candidate regions on CFA6, 9, 24, 27 and X, while candidate genes previously investigated in other species including *ESR1*, *NR5A1*, *GNRHR*, *HOXA10*, *HOXA11*, *FGFR1*, *SOS1*, *WT-1*, *INSL3*, *AMH*, *CALCA*, *PROKR2*, *LGR8*, *COL2A1*, *KAL1*, and *AR* were not significantly associated [13]. A more recent candidate gene study investigated polymorphisms in *MAMLD1*, *SRD5A2*, and *AR*, but an association with an increased risk of cryptorchidism was not found [14]. Another recent study reported two possible genetic biomarkers associated with canine cryptorchidism; hypomethylation of a single CpG site in the 5’-flanking region of *INSL3*, and a SNP in the 5’ flanking region of RXFP2 [15].

For this investigation a large-scale multi-breed genome-wide association study (GWAS) approach was used to investigate the genetics of cryptorchidism in the dog, using DNA samples collected by veterinarians at Banfield Pet Hospital^®^ locations across the United States and Mexico and through direct-to-consumer DNA testing in the United States.

## Materials and methods

### Samples

DNA samples were collected via commercial testing of Wisdom Panel^™^ Premium, Wisdom Panel^™^ Essential, Wisdom Panel^™^ Health and Optimal Selection^™^ retail products, and genetic testing performed as a part of Optimal Wellness Plans^®^ for puppies, through Banfield Pet Hospital branches (Vancouver, WA, USA). Samples were either collected through non-invasive cheek swabbing by dog owners or veterinary professionals or through blood sampling by a veterinary professional at a Banfield Pet Hospital in line with regulations governing diagnostic testing. Consent for use of DNA data in research was obtained through the client’s agreement with terms and conditions of DNA testing through Wisdom Panel. All samples originated from the United States or Mexico.

### Genotyping

DNA extraction from whole blood and buccal swabs was performed at GeneSeek Laboratories (Neogen Corporation, Lincoln, NE, USA). Genotyping was performed on a custom 100k Illumina Infinium XT SNP microarray (Illumina, Inc., San Diego, CA, USA), also at GeneSeek Laboratories. The microarray was designed and validated for use following the same protocol and principles as previously described [16, 17]. Microarray genotyping analyses were carried out following manufacturer-recommended standard protocols for the Illumina XT platform (Illumina, Inc.). Only samples achieving at least 97% genotyping call rate were included in the study.

### Clinical information

For DNA samples submitted directly for genotyping through Banfield clinics, data from genotyped dogs was directly linked with clinical records stored in the Banfield EMR (electronic medical record). For DNA samples collected and submitted by general retail consumers of Wisdom Panel products, data from genotyped dogs was linked with the Banfield EMR by anonymised cross-matching of pet and owner information, in accordance with personally identifiable information (PII) regulations. The EMR was then queried for dogs diagnosed with inguinal cryptorchidism.

### Inclusion criteria

A total of 3736 male dogs were identified with DNA data and a diagnosis of inguinal cryptorchidism. Both unilateral and bilateral inguinal cryptorchids were included in the study. In addition, a separate GWAS was also performed with 778 abdominal cryptorchidism cases (both unilateral and bilateral). Controls were defined as dogs not recorded as cryptorchid in the Banfield EMR, and over the age of 1.5 years. Controls were limited to male dogs. Breeds of dogs were determined using the Wisdom Panel breed detection algorithm. Using a family tree based approach, purebred dogs were defined as dogs with at least seven out of eight great-grandparents of a single breed, with PCA positioning consistent with other representatives in the reference database for the majority breed.

### Genotype analysis

A total of 94,878 variants were available for analysis. After filtering of SNPs with greater than 5% missing data, minor allele frequency < = 0.01 and removing samples with greater than 5% missing data, a total set of 91,428 SNPs remained. Genome-wide association study analysis was performed using a linear mixed-model approach performed in the software package GEMMA v0.98.1 [12] including a centered relatedness matrix. Principal Component Analysis (PCA) was performed using PLINK [18]. Allele frequencies for case and control sets and odds ratios were calculated using PLINK. All data analyses were performed within the Databricks cloud-based data analytics system. Manhattan and QQ plots were created using the R package qqman v0.1.8 [19], PCA plots were created using the R package ggplot2 v3.3.5 [20]. All reported genome locations are given based on the CanFam3.1 genome build.

### Ethics statement

Genetic analyses were carried out on DNA extracted from owner-collected, non-invasive cheek swab samples, or from blood/cheek swab samples collected at certified veterinary clinics in accordance with international standards for animal care and research. All dog owners provided consent for the use of their dog’s DNA sample for research purposes. As this study was purely based on data analytics, animal research ethics approval was not required.

## Results

### Genome-wide association analysis

**Fig 1:**
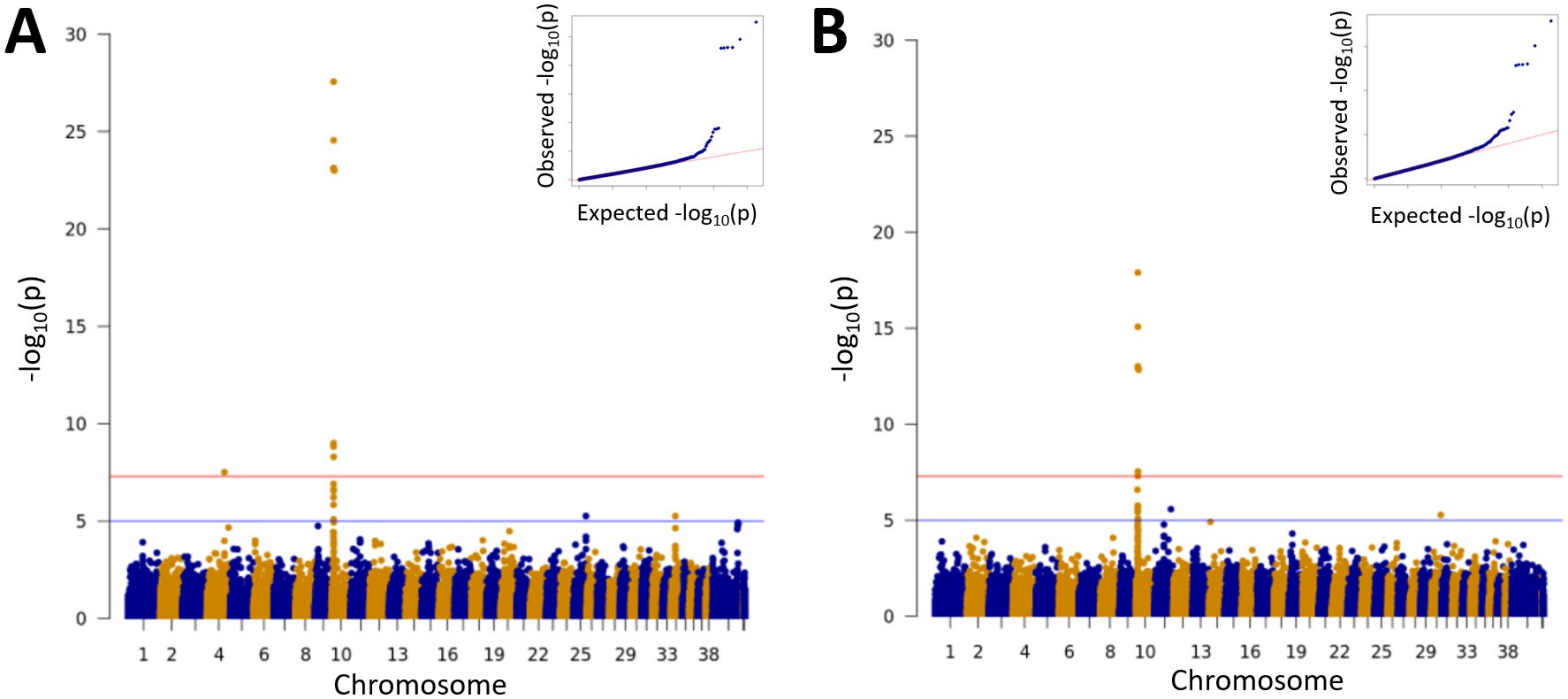
Inguinal cryptorchidism GWAS. (A) GWAS of 3736 inguinal cryptorchidism cases versus 3736 male controls. (B) GWAS of 3736 inguinal cryptorchidism cases versus 3736 genetically matched male controls to further correct for population substructure.

**Fig 2:**
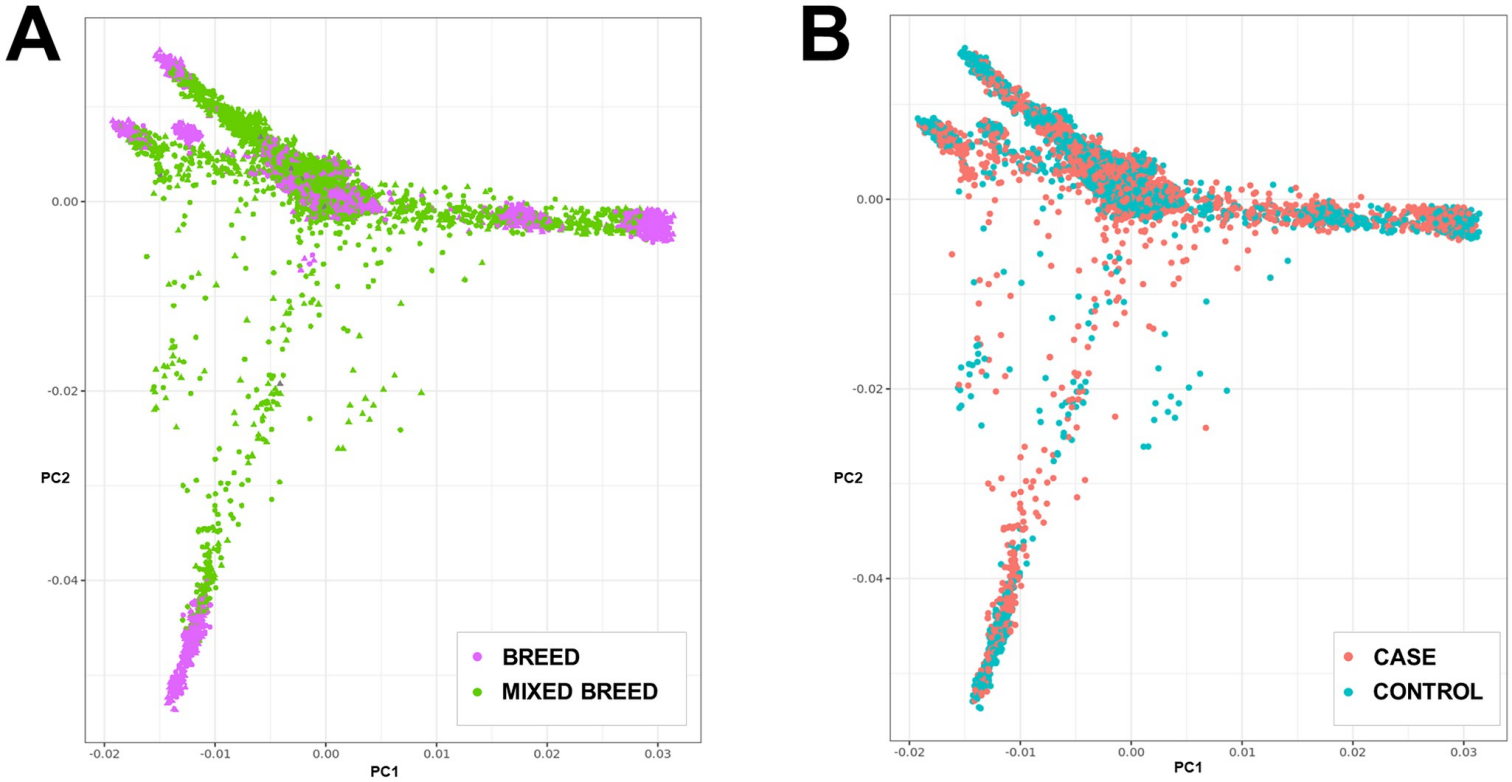
The complex genetic structure of the study sample set. (A) PCA showing the diversity of breed and non-breed individuals included in the study. (B) PCA plot showing the set of 3736 matched cases and controls.

Initial genome-wide association analysis was performed using the GEMMA software package on a dataset of 3736 inguinal cryptorchidism cases, and 3736 healthy male controls above the age of 1.5 years. A single peak on CFA10 reached genome-wide significance (Fig 1a). The top SNP (chr10:8348804; p = 2.77x10^-28^), located in the 5’ UTR of the *HMGA2* gene, was previously strongly associated with adult body weight [21]. As the case set consisted of a diverse combination of several breed and mixed-breed dogs (Fig 2), the GWAS was repeated with the same case set and an independent set of genetically matched controls. In brief, 3736 closely genetically matched controls were selected from a pool of 42,479 potential control individuals using the cluster function in PLINK with the cluster size set to two (one case and one control). The association on chromosome 10 remained (p = 1.27x10^-18^) (Fig 1b). A list of genome-wide significant SNPs can be found in S1 Table, which shows the top six SNPs to be all within or flanking *HMGA2*. A GWAS with 778 abdominal cryptorchidism cases and 778 controls revealed no signals reaching genome-wide significance (S1 Fig).

The genotype distribution of the top SNP is shown in Table 1. The odds ratio for individuals with two copies of the variant having cryptorchidism is 1.27 (p < 0.0001), compared with the genetically matched control set, which increases to 2.62 (p < 0.0001) versus the non-genetically matched controls.

**Table 1:** Distribution of *HMGA2* genotypes across cases and controls.

*HMGA2* genotype (chr10:8348804)	wt/wt	wt/var	var/var
**Cases (3736)**	1606	351	1748
**Controls—genetically matched (3736)**	1721	464	1532
**Controls—non-matched (3736)**	2218	544	940

Assuming a recessive model, the odds ratio of cases versus the matched control group is 1.27 (p < 0.0001), and versus the non-matched control group is 2.62 (p < 0.0001). Considering homozygous wildtype and heterozygous individuals (dominant model) the odds ratio of cases versus the matched control group is 0.811 (p = 0.0077), and versus the non-matched control group is 0.891 (p = 0.1287).

### Cryptorchidism and size

**Fig 3:**
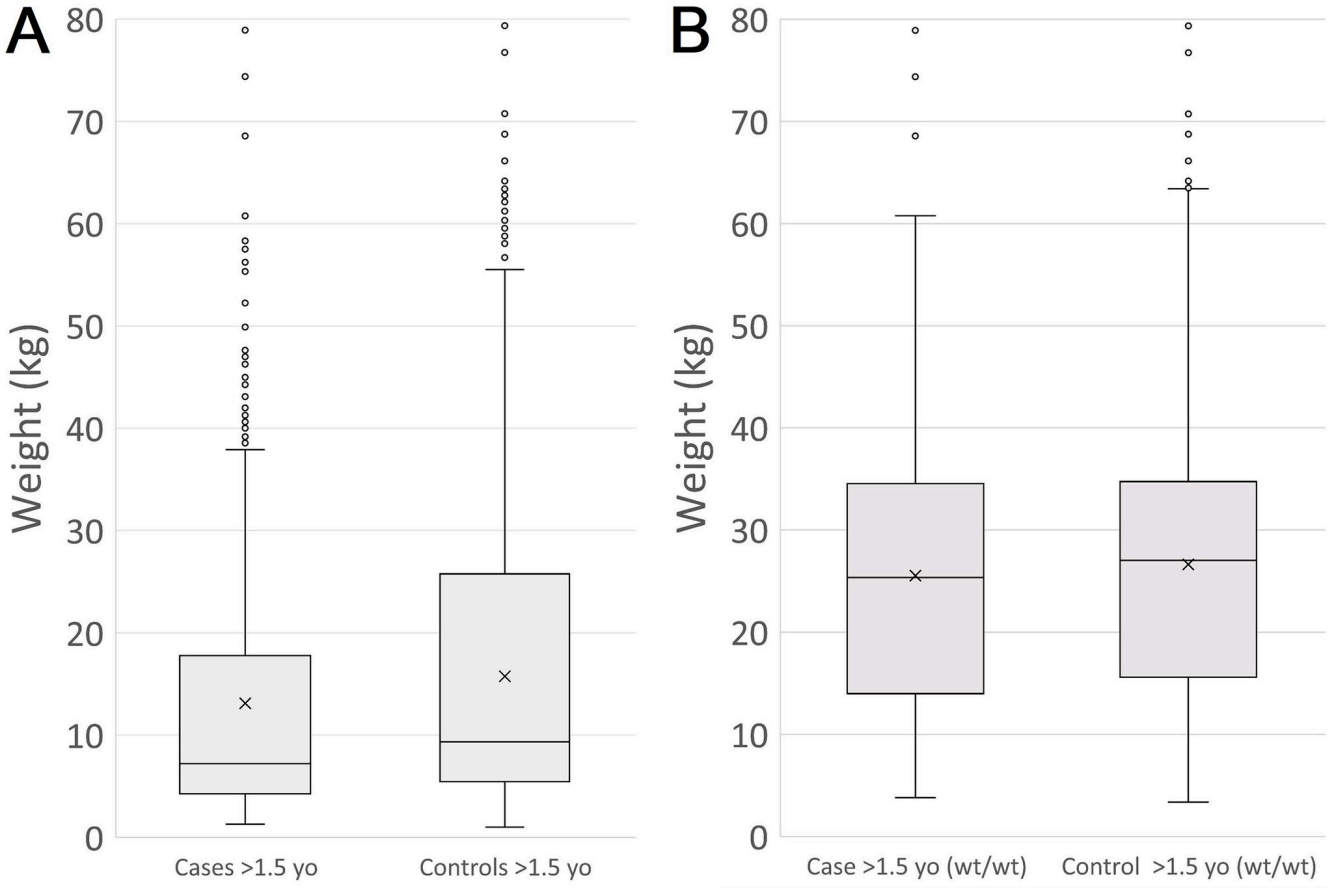
**(A)** Box plot representing the weight distribution of cases and genetically matched controls over the age of 1.5 years. The mean weight of cases (962) was 13.11Kg, compared with a mean weight for controls (3727) of 15.74kg (p = 1.198 x 10^−9^). **(B)** Box plot representing the weight distribution of *HMGA2* wt/wt cases and genetically-matched *HMGA2* wt/wt controls over the age of 1.5 years. The mean weight of cases (356) was 25.53kg, compared with a mean weight for controls (1718) of 26.63kg (p = 0.101).

As the *HMGA2* variant identified through GWAS was previously associated with adult weight, the weight distribution across cases and controls was investigated (limited to dogs over 1.5 years, with weight measurements recorded in the Banfield Pet Hospital database) (Fig 3). The mean weight of cases (962) was 13.11Kg, compared with a mean weight for controls (3727) of 15.74kg (p = 1.198 x 10^−9^; Kolmogorov-Smirnov (K-S) test). Excluding samples with copies of the *HMGA2* variant, the mean weight for cases (356) of 25.53Kg, compared with a mean weight for controls (1718) of 26.63Kg, was not significantly different (K-S test p = 0.101).

### Association analysis by breed

For breeds where at least 25 cases were available, individual within-breed GWASs were performed. Only association analysis in the Great Dane yielded a result of genome-wide significance (p = 9.18 x 10^−9^, CFA10:42,637,277), however this association was not in the region of *HMGA2*. An association just under genome-wide significance was found for the Shih Tzu breed (p = 9.18 x 10^−9^,CFA21:15,285,592). A summary of association results is shown in Table 2 and Manhattan plots are available in S2 Fig. Further analysis of association results within breed was outside the scope of this study.

**Table 2:** Summary of within-breed GWASs.

Breed	Cases	Controls	GEMMA Top SNP	Chr	Position	GEMMA (p)	λ
**American Staffordshire Terrier**	49	124	BICF2G630368222	23	45661039	5.64E-06	1.02
**Boston Terrier**	32	37	BICF2P1169231	31	35846921	6.88E-06	1.04
**Boxer**	44	52	TIGRP2P152285_rs8721801	11	66892546	2.62E-05	1.03
**English Bulldog**	53	64	TIGRP2P119557_rs9110060	8	64989168	1.32E-05	1.06
**Chihuahua**	56	77	BICF2P865353	27	9363932	3.06E-05	1.06
**French Bulldog**	154	180	BICF2G630645011	21	28071048	1.96E-06	0.99
**German Shepherd Dog**	97	181	BICF2S23310034	11	47723838	2.19E-05	1.03
**Golden Retriever**	43	53	BICF2P896811	11	19692283	1.05E-05	0.87
**Great Dane**	27	29	BICF2S23510683	10	42637277	9.18E-09	1.04
**Japanese Shiba Inu**	32	35	38_13861531	38	13861531	2.90E-05	1.03
**Labrador Retriever**	27	39	BICF2S23125293	6	67569529	3.39E-06	1.10
**Maltese**	43	70	BICF2P1129003	23	13340148	2.42E-05	1.01
**Pembroke Welsh Corgi**	36	36	BICF2G63052845	19	32261541	1.12E-05	0.94
**Pomeranian**	70	99	BICF2P774707	21	10493915	5.00E-06	1.00
**Poodle (Miniature)**	40	70	TIGRP2P276067_rs8859090	20	3956715	1.51E-05	1.02
**Pug**	65	89	BICF2P379588	14	46297901	1.42E-05	1.08
**Shih Tzu**	58	142	BICF2G630653470	21	15285592	7.60E-08	1.04
**Siberian Husky**	54	121	TIGRP2P18485_rs9026230	1	25874646	3.27E-06	1.01
**Yorkshire Terrier**	65	114	BICF2G630848023	16	16262588	2.09E-05	1.01

An association of genome-wide significance was found for the Great Dane, and an association of just under genome-wide significance for the Shih Tzu breed. Genomic inflation value (λ) is shown in the final column.

## Discussion

This investigation demonstrates the use of genotyping data generated through the process of commercial DNA testing, in combination with a veterinary EMR database, to perform a large-scale GWAS into canine Cryptorchidism. Results of the GWAS reveal a genome-wide significant association with the *HMGA2* variant previously associated with adult weight in dogs.

The high mobility group A2 (*HMGA2*) gene encodes a non-histone chromosomal and architectural transcription factor that facilitates changes in chromatin structure. HMGA proteins associate both with DNA and nuclear proteins, and are thought to act as part of the enhanceosome. HMGA has three AT-hooks that are highly conserved among HMGA proteins, allowing them to interact with the minor groove of DNA. The second AT-hook motif appears to be the most critical for protein function and is highly conserved among diverse organisms [22]. *HMGA2* expression is maximized in embryonic development, yet low in fully differentiated cells. Its expression in adult tissues has been associated with the formation of both benign and malignant tumors, and roles in adipogenesis and mesenchymal differentiation have also been suggested [23]. Overexpression of *HMGA2* in malignant tumors has been associated with a poorer prognosis and may be associated with cancer cells showing stem cell-like behaviors [24]. *HMGA2* variants have been associated with height, head circumference, intracranial volume, and permanent dentition [25]. *HMGA2* knockout mice show a pygmy phenotype consistent with the size reduction seen in dogs, and *HMGA1/HMGA2* double knockouts show a super-pygmy phenotype [26], characterized by reduced birth weight and growth retardation. Inactive *HMGA2* has been shown to result in smaller sizes and lower organ weights, as well as infertility and abnormal testicular descent [27]. In pigs, its deficiency has been associated with dwarfism, uneven fetal resource allocation, and cryptorchidism [22]. In one study, *HMGA2* deficient pigs were shown to be smaller in size than their non-mutant counterparts, their organ weights were reduced, and all of the *HMGA2* null/null individuals were sterile and cryptorchid [22]. A 1985 retrospective study looking at closely interrelated breed groups in the dog (e.g. toy, miniature, and standard Poodles) found that the risk of cryptorchidism in the smaller version of the breed was always greater than in its larger relation, suggesting that testicular maldescent could be related to size or growth rate [6], and lending credence to the idea that the *HMGA2* small size variant may be linked to cryptorchidism in the dog. Later studies confirmed that *HMGA2* was implicated in smaller body size in the dog [21], as well as possible behavior effects on separation anxiety, touch-sensitivity, owner-directed aggression, and dog rivalry [28].

Controlling for population stratification can be challenging when performing GWAS in the dog, with substructure seen even within breeds, potentially due to country specific subpopulations and the effects of line breeding and artificial selection. To thoroughly control for this, we used a dual strategy of using a mixed-model analysis approach and using genetically closely matched individual cases and controls in the investigation. Furthermore, as size had previously been linked with cryptorchidism risk, and because we identified that cases in our set had a significantly lower adult weight than controls, we took additional steps to confirm that the strong association of *HMGA2* with cryptorchidism was a true association rather than simply due to smaller dogs being selected as cases because of their increased risk of cryptorchidism. To investigate this the weight of adult cases and controls in the study without copies of the *HMGA2* disease-associated variant was assessed. The results showed no significant difference between the two groups, suggesting that *HMGA2* was driving the lower average adult weight of cases in the overall sample set, rather than adult weight being a generic risk factor for cryptorchidism. This ties in with the fact that several variants in many different genes have been strongly associated with adult weight in dogs, but only the variant in *HMGA2* was identified as a strong signal in this study. Interestingly a GWAS for abdominal cryptorchidism revealed no signals of genome-wide significance, suggesting that either 778 samples did not provide sufficient power to identify associated loci or that this form of cryptorchidism is due to a non-heritable clinical cause. While *HMGA2* was not sequenced for any of the cases or controls investigated in the study, as cryptorchidism has previously been associated with small size, we hypothesized that the variant found in the 5’ UTR of HMGA2 is having the dual function of influencing adult weight and increasing risk of cryptorchidism in dogs.

Within-breed GWAS for 19 dog breeds resulted in a single genome-wide significant association in the Great Dane breed. A replication study would be needed to further solidify this association. No breed produced significant results for *HMGA2*, probably due to the variant being fixed in a homozygous form for many of the breeds studied, or due to the modest increase in risk caused by the *HMGA2* variant. This further demonstrates that for variants that convey modest increases in risk, which is expected for many complex disorders in the dog, large numbers (thousands) of cases will be required to elucidate the risk factors for these conditions. It is also likely that to achieve these high numbers of samples, studies will need to be conducted across breed and mixed-breed populations. This is slightly contradictory to the view that the purebred dog population structure may help to unravel the genetics of complex disorders within the breed due to smaller effective population sizes with fixed and limited gene pools, although it is undoubtedly true that this population structure aids the mapping of autosomal recessive conditions in the dog.

In this study we have used integration of DNA data produced from a high-density SNP microarray with clinical records to define a large study set for GWAS analysis. This integration with ever expanding and regularly updated clinical records allows for the analysis of complex inherited disease at scale with the ability to identify risk factors, as demonstrated in this study. In time this approach will enable the development of polygenic risk scores which can be directly reported to the clinician, enabling personalised advice to be delivered to the client and an appropriate plan to be tailored for the individual dog.

## Supporting information

S1 Fig Manhattan plot of a GWAS with 778 abdominal cryptorchidism cases and 778 controls. (PPTX)

S2 Fig Manhattan plot for within breed GWAS of inguinal cryptorchidism in dogs. (PPTX)

S1 Table A list of genome-wide significant SNPs and their genomic coordinates. (XLSX)

S2 Table Sample IDs for cases and controls used in the study, with associated breed. (XLSX)
