# Supplementary material for: Large scale across-breed genome-wide association study reveals a variant in HMGA2 associated with inguinal cryptorchidism risk in dogs
Source: PLoS One. 2022 May 26;17(5):e0267604. doi: 10.1371/journal.pone.0267604 (PMC9135263; doi:10.1371/journal.pone.0267604)
Supplement: S1 Fig — (PPTX) [file pone.0267604.s001.pptx]

## Slide 1
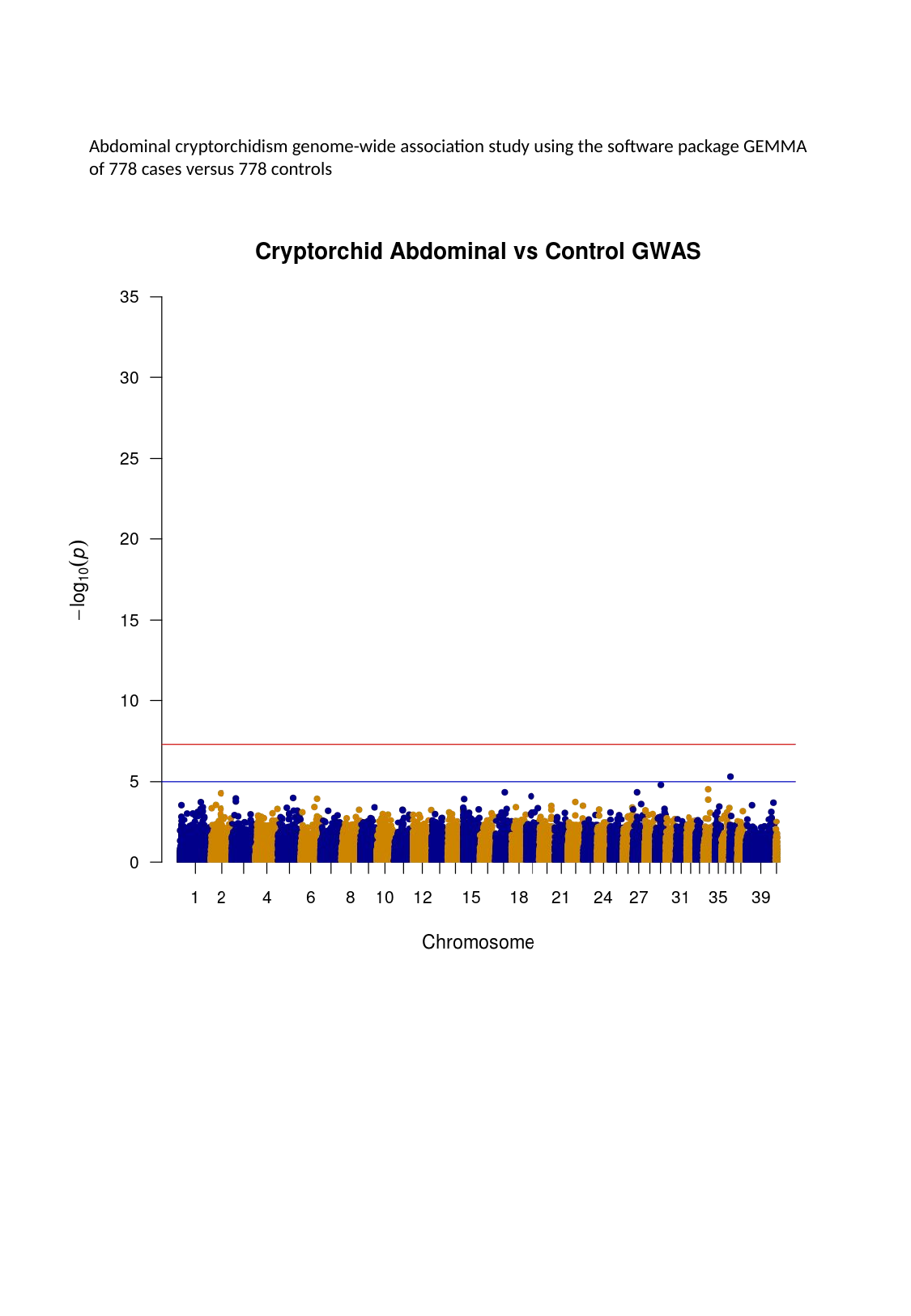

Abdominal cryptorchidism genome-wide association study using the software package GEMMA of 778 cases versus 778 controls
